# Using human factors principles to redesign a 3D lab workflow during the COVID-19 pandemic

**DOI:** 10.1186/s41205-022-00161-9

**Published:** 2022-11-12

**Authors:** Ethan P. Larsen, Elizabeth Silvestro, Daria F. Ferro, Asif Chinwalla, Natalie Oppenheimer, Sarah Rogers, Raymond W. Sze, Flaura K. Winston

**Affiliations:** 1 Department of Radiology, Children’s Hospital of Philadelphia, 3401 Civic Center Blvd, Philadelphia, PA 19104 USA; 2 Innovation Ecosystem, Children’s Hospital of Philadelphia, Philadelphia, PA USA; 3 Perelmen School of Medicine, University of Pennsylvania, Philadelphia, PA USA; 4 Division of General Pediatrics, Department of Pediatrics, Children’s Hospital of Philadelphia, Philadelphia, PA USA; 5 Department of Biomedical and Health Informatics, Children’s Hospital of Philadelphia, Philadelphia, PA USA; 6 Kellogg School of Management, Northwestern University, Evanston, IL USA; 7 McCormick School of Engineering, Northwestern University, Evanston, IL USA

**Keywords:** 3D printing, COVID-19, POC manufacturing, Human factors engineering, Quality improvement, Work systems, Additive manufacturing

## Abstract

**Background:**

Like most hospitals, our hospital experienced COVID-19 pandemic-related supply chain shortages. Our additive manufacturing lab’s capacity to offset these shortages was soon overwhelmed, leading to a need to improve the efficiency of our existing workflow. We undertook a work system analysis guided by the Systems Engineering Initiative for Patient Safety (SEIPS) construct which is based on human factors and quality improvement principles. Our objective was to understand the inefficiencies in project submission, review, and acceptance decisions, and make systematic improvements to optimize lab operations.

**Methods:**

Contextual inquiry (interviews and workflow analysis) revealed suboptimal characteristics of the system, specifically, reliance on a single person to facilitate work and, at times, fractured communication with project sponsors, with root causes related to the project intake and evaluation process as identified through SEIPS tools. As interventions, the analysis led us to: 1) enhance an existing but underused project submission form, 2) design and implement an internal project scorecard to standardize evaluation of requests, and 3) distribute the responsibility of submission evaluation across lab members. We implemented these interventions in May 2021 for new projects and compare them to our baseline February 1, 2018 through – April 30, 2021 performance (1184 days).

**Results:**

All project requests were submitted using the enhanced project submission form and all received a standardized evaluation with the project scorecard. Prior to interventions, we completed 35/79 (44%) of projects, compared to 12/20 (60%) of projects after interventions were implemented. Time to review new submissions was reduced from an average of 58 days to 4 days. A more distributed team responsibility structure permitted improved workflow with no increase in staffing, allowing the Lab Manager to devote more time to engineering rather than administrative/decision tasks.

**Conclusions:**

By optimizing our workflows utilizing a human factors approach, we improved the work system of our additive manufacturing lab to be responsive to the urgent needs of the pandemic. The current workflow provides insights for labs aiming to meet the growing demand for point-of-care manufacturing.

**Supplementary Information:**

The online version contains supplementary material available at 10.1186/s41205-022-00161-9.

## Introduction

The COVID-19 pandemic forced all areas of healthcare to adapt to maintain safe operations [1]. In the spring of 2020, the availability of personal protective equipment (PPE) for respiratory precautions and other care-related supplies became limited [2, 3]. Institutions facing supply chain disruptions sought alternative strategies to maintain adequate supplies, including reconditioning used equipment and employing additive manufacturing (3D printing) to provide alternate solutions where possible [4, 5].

The Children’s Hospital of Philadelphia (CHOP) Department of Radiology supports an in-house additive manufacturing resource, the Children’s Hospital Additive Manufacturing for Pediatrics (CHAMP) Lab, which serves the entire hospital and handles design, rapid prototyping, and 3D printing of clinical and research devices, surgical models, and training tools [6]. The supply chain shortages caused by the COVID-19 pandemic placed new pressure on the CHAMP lab to help fill gaps in supply chain shortages. In addition to our existing project workload, the CHAMP lab was called on to produce backup supplies for ventilators, nasopharyngeal swabs, and personal protective devices. Nearly all requests were urgent, and for some, specifications changed after project initiation because of uncoordinated or inconsistent communication from stakeholders and shifts in material availability. In one instance, CHAMP offered to produce face shields and was told immediately that those services would not be needed. A few weeks later, a different representative from the same group sought out CHAMP with an urgent request for five times as many face shields as originally offered. Incomplete submissions for urgent projects necessitated multiple emails to clarify information and added burden to the lab. CHAMP realized addressing the additional requests while meeting existing research demands was straining the lab’s resources and ability to manage its workload. This mirrored a global surge in 3D printing to meet pandemic needs seen in hospital/university labs, community efforts, and local manufacturing [4].

In August 2020, we undertook an analysis of the lab’s operations during the pandemic in collaboration with the CHOP Innovation Ecosystem (IE), a multidisciplinary group that seeks to foster innovation through collaboration with cross-functional teams working to develop and enhance institutional innovation. In that analysis, based on interviews with lab members and observation of lab functioning, *workflow* was identified as a key opportunity for improvement [5]. The IE’s analysis separated the lab’s workflow into 3 aspects: project submission/intake assessment, design selection, and production/delivery. The pandemic conditions demonstrated the need for the lab to be more responsive to demands and to document an initial list of requirements and associated information from the right stakeholders from the initiation of a project. We sought to address these needs with the assumed constraint of a hiring freeze that required completion of added pandemic-related work without an increase in staffing.

Many institutions, including ours, turned to new-to-healthcare skillsets like human factors (HF) engineering to address their rapidly evolving needs during the pandemic [7]. With a broad spectrum of skills, HF has effectively informed rapid response to large system changes [8]. One facet of HF, sociotechnical work systems (STS) theory, focuses on the complex interactions of the components of the work system, and holistically examines them to find optimized solutions for sub-optimal systems [9–11]. HF tools have also been adapted to the area of patient safety [12, 13]. One specific framework that applies STS and leverages quality improvement (QI) principles is the Systems Engineering Initiative for Patient Safety (SEIPS) [9, 10], an investigative framework that uses a whole system perspective to scope work systems and the interaction of system components with the aim of providing design criteria for interventions. The use of SEIPS has demonstrated improvements in primary care processes [11], workflows to increase patient safety [9], and new approaches to improve medication safety [14].

When QI interventions are introduced under consistent conditions, sequential evaluations can measure incremental changes toward a desired goal. The pandemic scenario, however, was and continues to be far from stable with demands and procedures changing constantly as demands and knowledge evolve. Further, reaction to the pandemic conditions were far from incremental, at times requiring drastic and comprehensive action. Responding to these highly variable conditions is where whole system perspectives like SEIPS are particularly useful [9, 11]. We chose SEIPS as the appropriate framework for the current scenario based on prior literature demonstrating the success in utilizing SEIPS for environment changes, workflows for patient care, and telemedicine [7, 15]. Further, given the unpredictable nature of a pandemic, SEIPS was well-suited to the work due to its comprehensive perspective, rapidly deployable framework, and adaptability. To our knowledge, this is the first expansion of the application of SEIPS beyond patient safety to work systems in a point-of-care manufacturing lab. The expansion into the domain is significant as small additive manufacturing labs like CHAMP are growing in popularity, and those that are in place are being challenged with increasing demand due to current variability in supply chain capabilities.

As part of a larger effort, we sought to conduct a SEIPS-based evaluation of the existing CHAMP lab workflows and provide design criteria for interventions to enable the lab to respond to pandemic-related and other product demands and provide preliminary evidence of its impact. Many of the issues identified originated with aspects of project intake and early actions/decision-making. The desired outcomes were to improve efficiency (the speed) and throughput (completion) by: (1) improving and standardizing the project intake and selection process, (2) identifying and maintaining consistent communications with stakeholders, (3) reducing lab reliance on a single individual to move project requests along, and potentially (4) reducing the time to reach a decision on project acceptance. The work detailed in this manuscript conforms to the SQUIRE 2.0 guidelines for the reporting of QI projects [16].

## Methods

### Context

CHOP launched its 3D printing program in 2011. In 2016, the program officially became the CHAMP Lab. The CHAMP Lab is led by a Lab Manager, a Masters-trained mechanical engineer (ES), and Lab operations are supported by a team of radiologists, physicians, technologists, researchers, and other radiology department personnel. With the exception of the manager and two 3D technologists, the remaining members of the CHAMP Lab team divide their time between the Lab and other responsibilities. The projects carried out by the CHAMP Lab are broadly designated as surgical, education, research, devices/tools, or training/phantoms, and are assigned to different “product streams” for production. Clinical requests (e.g., surgical models), which were out of scope for this project, are coordinated separately and handled through electronic health record (EHR) order sets to preserve HIPAA compliance. Prior to the onset of COVID-19, the CHAMP lab handled a total of 79 documented projects dating back to 2018.

### Planning the interventions

An investigative team from the IE set out to help the CHAMP lab apply the SEIPS framework to examine the handling of burgeoning workload including the intake process during the pandemic. The IE investigative team was comprised of specialty researchers in areas of HF, informatics, QI, and medicine (AC, EL, DF, NO, RS, FW). With input from CHAMP lab member interviews, the investigative team mapped the workflow and practices of the lab through SEIPS tools. The first tool, a task matrix, lists key tasks and identifies details such as how tasks are performed, the individuals who typically perform the task, and the goals of the tasks [10]. Similarly, a tools matrix assesses the users, purpose, and usability of the tools that facilitate the tasks. The SEIPS tools allowed the team to systematically deconstruct elements of the workflow (both pre-pandemic and early in the pandemic) and identify opportunities for potential improvement.

Through the creation of a task matrix (Table 1), it became apparent that the CHAMP Lab Manager bore the responsibility for moving all work forward across the entire workflow. Concurrently, examination of the tools matrix (Table 2) demonstrated that the skills and knowledge necessary for these steps were not exclusive to the Lab Manager and would be attainable for most members of the Lab. In addition, before the pandemic, projects frequently stalled waiting for changing demands from stakeholders. These struggles were exacerbated by increased demands for COVID-related products. Further investigation into the workflow of the CHAMP Lab uncovered common themes related to difficulties when project stakeholders changed or request parameters were altered mid-project, ultimately leading to communication between the CHAMP Lab and the project initiator becoming fractured. Root cause investigation into these challenges revealed a common driver, the initial project submission. Therefore, based on effort vs impact analysis, the team focused interventions around improving the intake submission process.

**Table 1:** CHAMP Lab Task Matrix (pre-intervention)

Task	Who Performs	Goals	Frequency	How Performed	When Performed
Request Evaluation	Lab Manager	Determine if project request fits with lab goals	Requests reviewed weekly	Subjective review of submitted responses	As requests arrive
Request completeness check	Lab Manager	Determine if all necessary information is present	Completed with Request Evaluation	Review of responses to ensure completeness	As requests arrive
Project initiation	Lab Manager leads group discussion	Search for existing products and designs that may address need	After request approval	Group vote at team meeting	Weekly meeting after request has been evaluated
Product Design	Lab Manager	Engineering design of desired product	Iterations as needed to refine design	Independently by lab project lead	When possible after project accepted
Printing	Lab Manager	Creation of engineered product	As needed	Printing handed off to printer trained personnel	After product design completion
Stakeholder Review	Lab Manager	Stakeholder approval of design	At least once per project	Coordinated by project lead, other members as needed	After printing prototype completed
Final Delivery	Lab Manager	Handoff of product and project conclusion	Once per project	Interdepartmental mail or direct handoff	Upon completion of final product printing

**Table 2:** CHAMP Lab Tools Matrix (preintervention)

Tool	Users	Purpose	Frequency	Ease of Access	Usability
Online submission form	External submitters	Provide unified point of contact for project intake	Intakes push to project board	Anyone with URL can submit	Guided computer based survey
Project Board	Lab Team	Organize projects and lab productivity	Weekly review	Registered users	Click and drag interface, relatively intuitive
Tazbot	Trained Lab members	FDM Printer	As needed for projects	Physical access required	Device specific training required
J750/Objet 30		Polyjet Printers			
Formlabs		Sterilizable Material printer			
CAD	Trained lab members	Create printable models	Needed for all projects	Adequate free options exist	Basic education required

While SEIPS can inform improvement interventions, it also can identify aspects of a process that should be preserved and bolstered. One such aspect was the existing use of a CHAMP lab shared project management board, Trello [17], to facilitate weekly lab meetings. Using the Trello board allowed all members to check in with updates to their projects and be asked for input on other Lab work. The structure of the board created a living agenda for weekly team meetings, minimizing administrative work and theoretically allowing anyone to facilitate the meeting.

### Interventions

#### Role responsibility

The first SEIPS-derived intervention in creating a shared CHAMP lab workflow was to propose new role distribution such that, where appropriate, tasks did not solely rely on the Lab Manager. Many of the typical tasks in the project management process could be accomplished collaboratively and with shared responsibility by a project leader in conjunction with the Lab Manager and support from the rest of the team.

#### Project submission form

Although a formal submission portal was available online, project requests had often been made through email and direct communication to the Lab Manager. Pre intervention, only 66% (*n* = 52) submissions were made through the intended portal. Other submission mechanisms included in person (*n* = 5), direct email (*n* = 15), and 7 projects where submission data were not available. Working with an external consultant, we developed and tested a new public project submission form (see Additional file). Recent project initiators were asked to voluntarily resubmit their intake request on the new system and complete a brief survey comparing their experiences. The new project submission form was iteratively improved based on this feedback and to align with CHAMP Lab work.

#### Scorecard

**Fig. 1:**
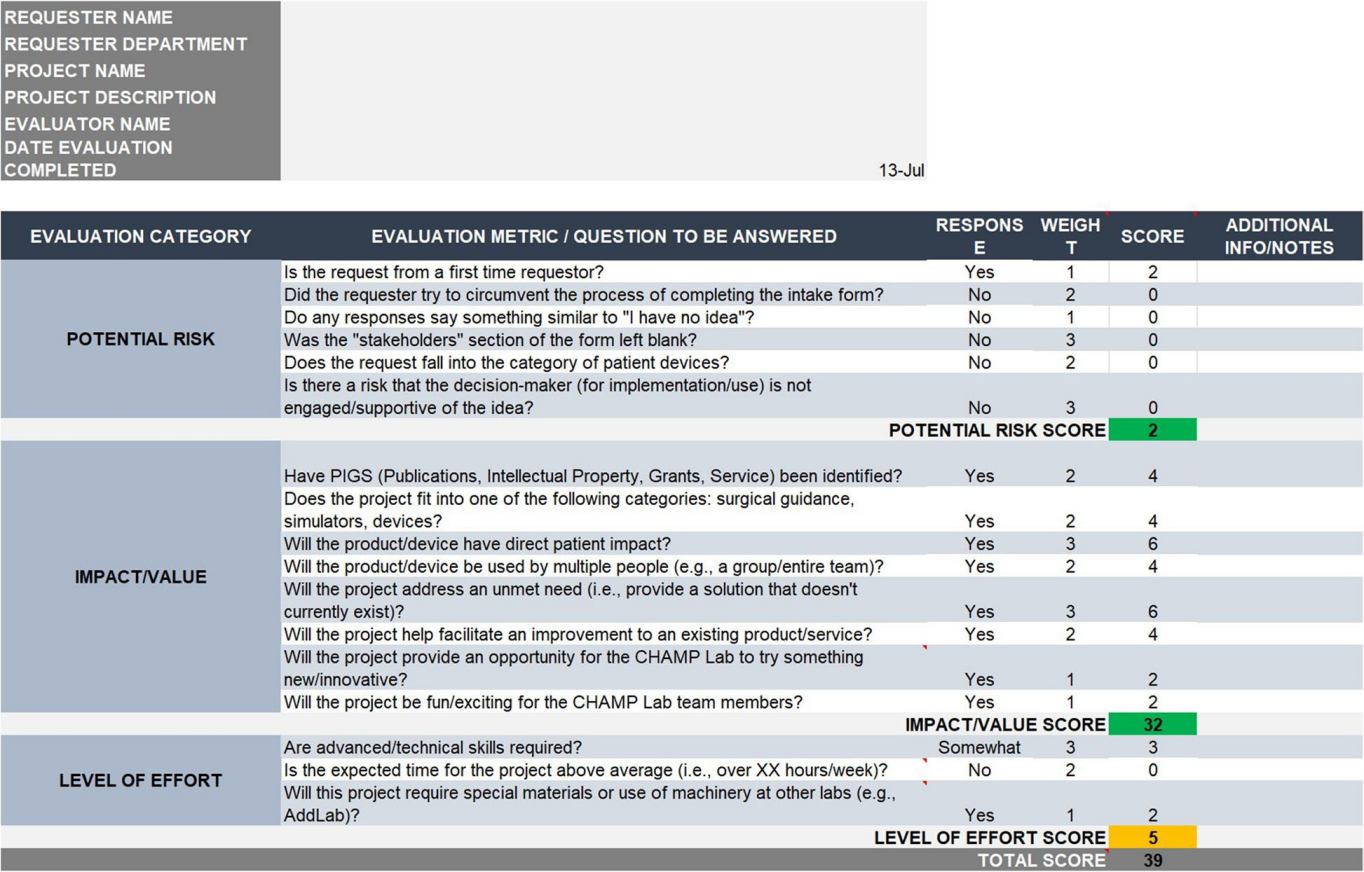
Internal Project Evaluation Scorecard

During the submission form redesign, the consultant also worked iteratively with the CHAMP lab to design and implement an internal project scorecard to be used in evaluating new project requests (Fig. 1). Previously, projects were reviewed solely by the Lab Manager and communication back to the requester was not systematic with some projects never receiving an initial evaluation. The scorecard contains a list of metrics/questions to assess the potential risk, value, and level of effort associated with a project. Simple pull-down menus in the scorecard are equated to numerical values so all lab members grade to the same scale. The questions were developed through input from the CHAMP lab staff based on prior experiences. The structure of the scorecard is such that any user is able to make a comparable assessment of the potential value of a project and lead the discussion.

Both the submission form and scorecard were designed to assess key productivity measures of the CHAMP lab, namely peer-reviewed manuscripts, new intellectual property, external funding, and provision of service to clinical operations and research. In addition, the project submission form prompts the user to consider if there are alternate products that may serve their needs. This approach allows problems to be solved without unnecessarily taxing CHAMP resources; while 3D printing is a solution to a problem, it is not always the only or best-suited solution.

**Fig. 2:**
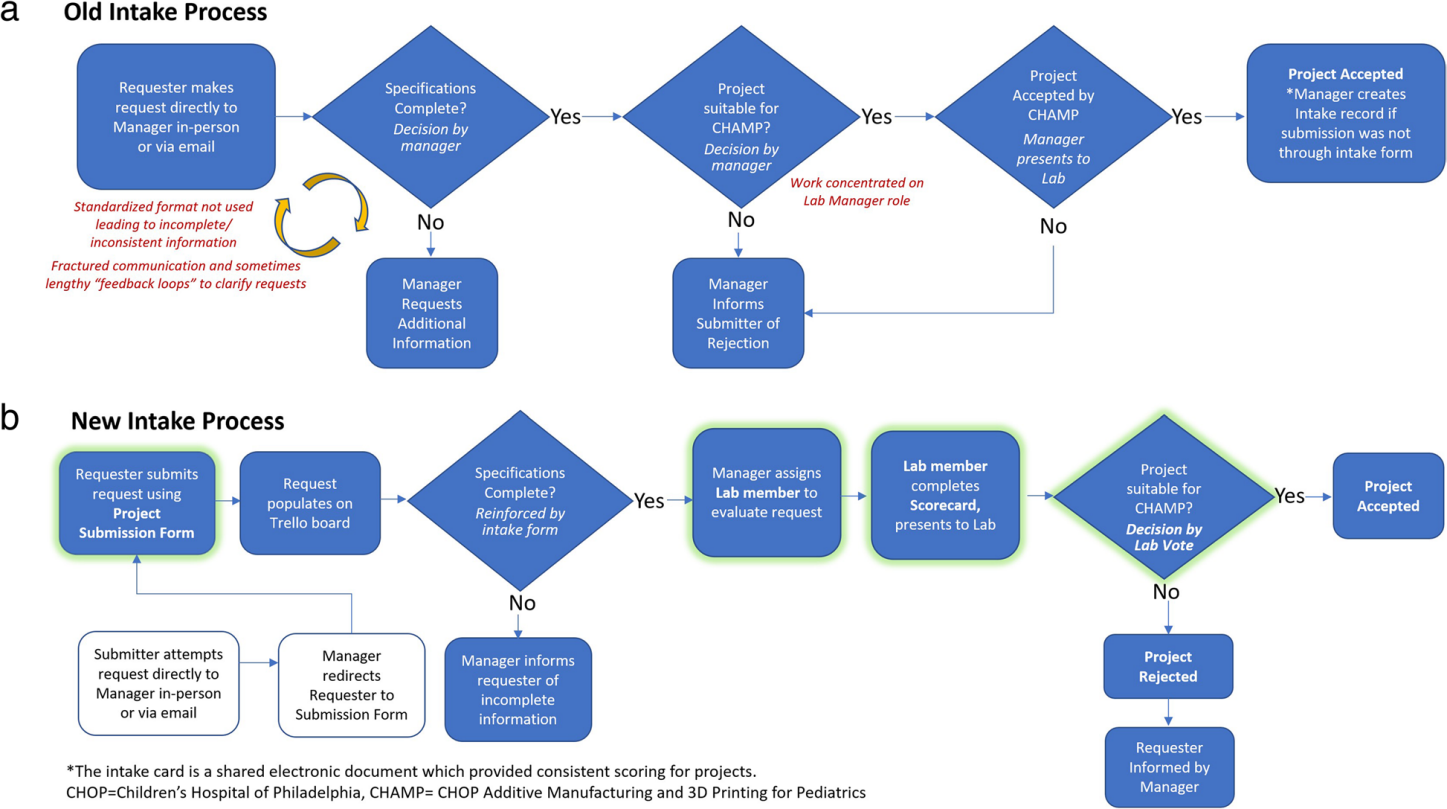
**a, b** Old and New Intake Process Workflows (paneled)

The redesigned submission and evaluation process (Fig. 2a and b) involves the requester using the new online project submission form to submit their project. Requests are accepted only through the designated submission form; all emails, phone calls, and direct communications attempting to submit projects are redirected. Both the original and improved submission form directly populate an intake queue on the Trello board, helping to ensure that projects are assessed promptly and regularly. The “Request” section of the Trello board is reviewed regularly by the Lab Manager and any incomplete submissions are referred back to the submitter by the Lab Manager. The Lab Manager then assigns complete submissions to a CHAMP Lab member for evaluation. Typically, request evaluation assignments are made sequentially to distribute work unless a project seems to particularly align with a Lab member’s skills. The assigned Lab member evaluates the submission using the scorecard and, if necessary, looks into current conditions or available equipment. The completed scorecard automatically generates and color codes the project in categories of risk, impact/value and expected level of effort. At the next weekly Lab meeting, the reviewing member presents the scorecard and project for a Lab vote on “accept” or “reject’. After the Lab has reached a decision, either the Lab Manager or reviewing member contacts the project submitter with the decision and initiates the project. Submitters whose projects are rejected are informed of the decision rather than potentially indefinitely deferred for more revisions.

The member who completes the scorecard will typically act as the project lead and coordinate subsequent activities with the Lab Manager and stakeholders until final product delivery. They are also responsible for reporting regularly at CHAMP Lab meetings about project progress. The Lab Manager utilizes the Trello board as a living agenda to facilitate Lab meetings. The individual Trello cards are updated by the manager with notes during weekly meetings in place of separate minutes. While it has not been necessary, the project scorecard is also intended to provide some measure for project prioritization when multiple projects have similar deadlines.

### Studying the intervention

We implemented the above interventions for all new project requests starting in May 2021 and monitored performance in the CHAMP Lab through May 2022 (365 days). The old submission form was deactivated, and all URL links updated to reflect the new form at that time. The existing Trello board tracked engagement of lab members in the project intake process through use of the scorecard and provided our archival record of projects. Perceptions of improvement by the CHAMP Lab team were qualitatively assessed through informal discussion with the Lab team during a CHAMP meeting. The pre-intervention period was defined as February 1, 2018 through April 30, 2021 (1184 days) which represents the duration which the original intake mechanisms (TypeForm, email, and direct communication) were active.

### Measures

Changes in CHAMP Lab performance from the interventions were observed through several metrics. As process measures, we tracked the proportion of requests that used the new project submission form as well as the proportion of requests that were evaluated using the scorecard and the proportion of Lab members who led an evaluation using the scorecard. We also tracked the number of project requests that received definite responses for “accept”, “reject”, or “more information needed” decisions. Finally, we assessed CHAMP team perceptions of project management under the new intake workflow. As impact measures (measures of improvement), we tracked throughput to successful completion (# completed projects/# total intakes) and termination of projects that were not progressing (# projects in the stalled state) in addition to time to review new submissions (# days to review).

### Data collection and analysis

Pre-intervention data were collected by retrospective retrieval of time stamps from the prior mechanisms used by the CHAMP lab (email, original submission form, Trello cards). Post-intervention data were extracted from the new project submission form, Trello, and calendar invitations. Data collected related to the dates a project was initially submitted and delivered. In addition, when available, dates of an intermediate planning brainstorm activity, and CHAMP Lab review were also captured. Where available, equivalent measures of time and number of projects are compared between the pre intervention (February 2018 to April 2021) and the post intervention (May 2021 to May 2022) phases.

### Ethical considerations

This project was undertaken as a Quality Improvement Initiative and as such does not constitute human subjects research.

## Results

**Fig. 3:**
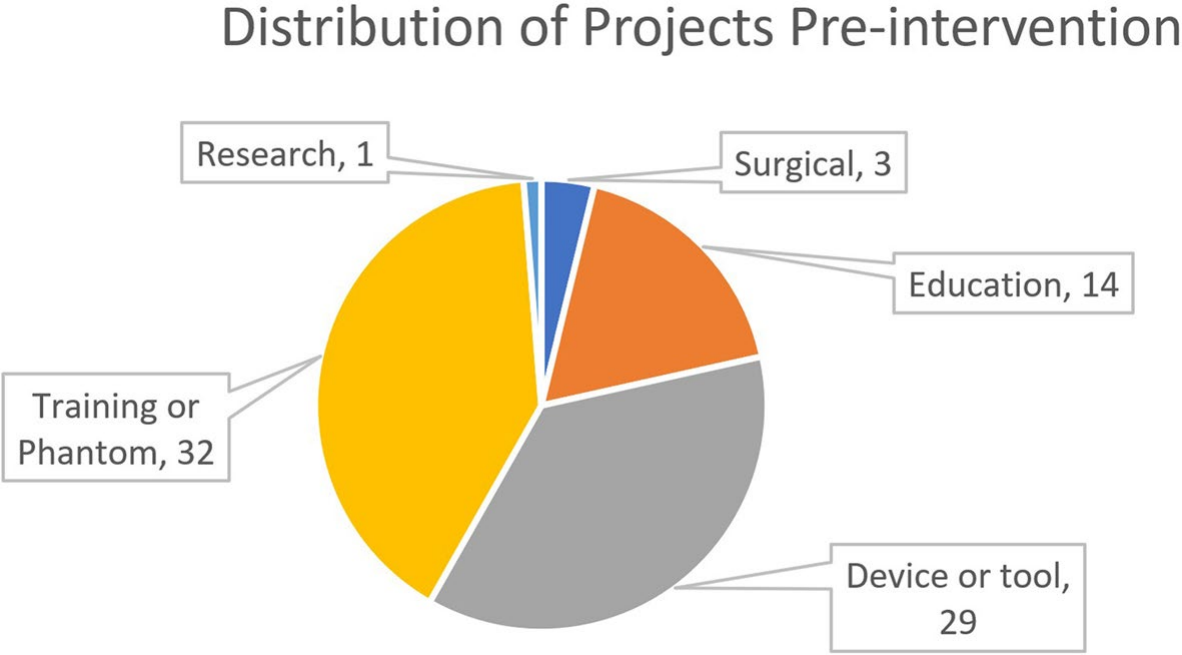
Distribution of CHAMP Projects Pre Intervention

In the pre-intervention state, the CHAMP Lab had handled 79 projects (between February 2018 and April 2021), averaging 15.8 per year, with a peak of 23 projects in 2020 due to pandemic demands their distribution is presented in Fig. 3.

**Fig. 4:**
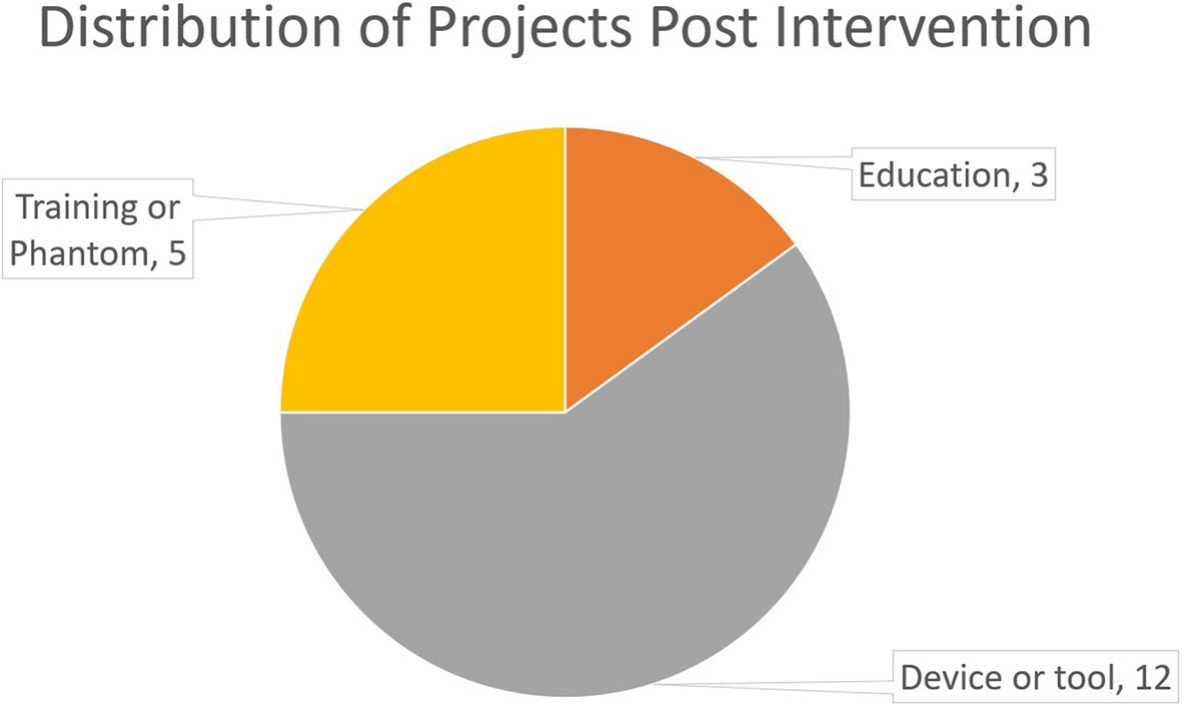
Distribution of CHAMP Projects Post Intervention

Since implementing the changes in responsibilities and workflow on May 1, 2021, the Lab has successfully evaluated 20 project requests over a 12-month period, the breakdown of projects is depicted in Fig. 4.

All 20 project requests used the new online project submission form (with none coming through email) as compared to the pre-intervention state which had only 66% (*n* = 52) of submissions made through the submission form. All projects were evaluated using the project scorecard, with all seven members of the Lab successfully evaluating at least one project and presenting their review to the Lab for a final decision. The Lab Manager has overseen the review and continuing efforts of 50% (*n* = 10); the remaining projects have been distributed amongst the other members of the CHAMP Lab. Of the 20 requests submitted since interventions, all received definite responses, 12 (60%) have been completed and delivered, 7 are in progress, 1 was rejected, and no projects are in a “stalled” state. This completion rate (throughput) was higher than the pre-intervention period where 35/79 (44%) of projects were completed, 24 are in a “stalled” state, and 8 projects were completed by the Lab but never implemented by requestor, and 1 was routed to an alternative solution. All requests received a scorecard-based review by a Lab member within 4 days; accepted projects were invited for a brainstorm session on average by 18 days from submission. Pre-intervention requests took an average of 54.8 days to receive a decision.

With the project submission form, we were also able to obtain necessary information, including identifying key stakeholders, earlier in the project. Based on review of the original submission form, critical fields in the new form are designated as “required”. However, in some cases the response of “I don’t know, I need help” is acceptable and informs the CHAMP Lab. Instances where the submitter indicated “I don’t know” are clear and can be factored into the decision-making process. The standardized evaluation criteria operationalized by the scorecard enabled us to make decisions, including decisions to reject a request, more quickly than we previously had. As a result, project requests receive a response from the CHAMP lab within 1 to 2 weekly CHAMP lab meetings. The new decision-making structure allows for any member of the Lab to manage project discussions; hypothetically, even interns can run this process.

Staff reaction to the new process was overall positive. Members were particularly focused on the new standardized scorecard, indicating that the scorecard gave all members an ability to consistently assess submissions for suitability and present them to the team in addition to accountability for tracking decisions. Reinforcement of the single venue for project submissions allows all lab members to easily recommend submission when they encounter CHOP personnel with a project that may work as a CHAMP project. Finally, the acceptance/rejection decision involves a more active discussion amongst the group, with talking points originating from the scorecard and defined assessment to inform requestors.

## Discussion

A SEIPS-guided redesign of a hospital-based 3D printing workflow during the pandemic produced improved productivity (requests managed per year pre: 15.9/year; post: 20) and higher completion rate (pre: 44% vs. post: 60%), allowing the lab to meet demands without an increase in staffing. This impact was driven by the successful improvement of the project submission and acceptance process. The largest benefit of the improved workflow for the CHAMP lab is that the scorecard now provides clear decision criteria for the acceptance or rejection of a project; projects that do not fit within the goals and critical criteria of the CHAMP lab are identified and rejected. The resulting impact is that time for a project to receive a decision dropped from 54.8 days to just 4. Prior to the intervention, poorly-defined and ill-suited projects could be caught in a feedback loop of requesting additional information and resubmission taking attention from other projects. This is demonstrated in the present state where no projects have been able to fall into the “stalled” category where they are not completed but not canceled either, as compared to the pre-intervention state where 24 projects have been stalled. Also, importantly, the submission form forces users to provide critical information in order to submit, preventing incomplete submissions, while allowing them to select “I don’t know” so the CHAMP lab can assist. In addition, the new workflow enables any member of the Lab to conduct an initial evaluation and present the project to the CHAMP team for a group decision. Typically, the individual who completes the initial scorecard can become the point of contact for a project, interacting with the stakeholder and relaying information to the lab. This distribution of responsibility reduces the burden on the Lab Manager to facilitate every aspect of all of the ongoing projects. Thanks to established shared responsibility for tasks, work flows through the lab more efficiently and does not come to a halt when the Lab Manager is unavailable or overburdened. Keeping consistent communication with the stakeholder also ensures that the products match the expectations and criteria of the stakeholder, even if the criteria may shift during the lifespan of the project.

The STS work in evaluating the intake process has resulted in the unification of the thought processes in project assessment. The scorecard was purposely designed to measure the key elements of interest by the CHAMP lab regardless of which team member was completing it. Another key benefit of utilizing an STS-driven improvement and intervention design process is that the interventions are tailored to fit the work system. For example, for the project scorecard, no additional ramp-up or training of lab members on its use was necessary due to its iterative development with the CHAMP team.

Similar to prior work conducted with SEIPS [11], we have successfully evaluated and made impact to a workflow. Though we were not concentrated on a direct patient impact, we were able to take a work system that was experiencing new stress from the COVID-19 pandemic and utilize human factors in another capacity to make improvement [7, 15]. Making use of the recently published SEIPS 101 materials, we were able to assess the current state of the work system and identify areas where workload could be shared [10].

The project was not without limitations. Most of the investigative work was conducted virtually due to evolving restrictions of the pandemic. Many of our pre/post comparisons are drawn from data collected through retrospective email review. Due to migrations of data across multiple Trello boards, many of the date-based metrics in Trello are approximate, relying on email time stamps as our primary data. Direct comparison of conditions were challenging throughout the pandemic due to supply chain circumstances shifting, most notably in measuring the complexity of the projects pre- and post-intervention. However, we feel that the evaluation and changes to the intake process collectively help the CHAMP lab to be more responsive and adaptable while continuing to produce projects of value. Despite the limitations, the pre- and post-intervention CHAMP lab personnel were unchanged (in the number of full-time equivalents and the people involved) and they felt that the projects pre and post were of similar complexity. Future work should include measures of project complexity (total effort in terms of total segmentation time and print time, financial costs and/or material volumes consumed). While balancing measures were not formally tracked, the CHAMP team feels that the changes to the intake workflow have improved overall performance. The redistribution of efforts has not noticeably altered meeting duration, and projects continue to move through the lab workflow appropriately.

Looking beyond the scope of this project offers a few potential applications of a similar process to other labs, workflows, and teams. Most importantly, the CHAMP lab will apply this approach to projecting the future growth of point-of-care manufacturing, using the metrics to project growing demand and needs to expand capability and adapt the process.

Beyond 3D printing, a SEIPS-based approach, tools, and process outlined in this paper could benefit any small, hospital-based service groups (e.g., biomedical engineering, human factors or quality assurance) that support various groups across a medical network in request-based format and face growing demand in the context of restrained staffing and resources. Several national efforts, such as the Radiological Society of North America 3D Special Interest Group and Society for Pediatric Radiology Quality and Safety Committee, exist to support and connect such groups and could be a potential space for sharing methods and findings.

## Conclusion

Deploying the SEIPS toolkit for QI facilitated a comprehensive evaluation of how work was performed in our hospital-based additive manufacturing lab? under the stressors of the early days of the pandemic. The drastic shift in situation and demands caused by the pandemic required an improvement approach that could scope the entire situation and propose changes that were responsive to the new landscape. Human factors methods are capable of providing the kind of response needed in developing fields such as additive manufacturing, alongside other traditional approaches.

Though motivated by the COVID-19 pandemic, our efforts to improve lab operations were certainly necessary and have been shown to be successful. As more hospitals seek to launch their own additive manufacturing labs and point-of-care manufacturing, we hope that other institutions learn from our experience and jumpstart their lab’s development and project management process without needing an industry-wide crisis to motivate change.

## Supplementary Information

**Additional file 1.** Project Submission Form.

## Data Availability

Data sharing is not applicable to this article as no datasets were generated or analyzed during the current study.

## Abbreviations

HF: Human Factors
STS: Sociotechnical work systems
SEIPS: Systems Engineering Initiative for Patient Safety
QI: Quality improvement
CHAMP: Children’s Hospital Additive Manufacturing for Pediatrics
CHOP: Children’s Hospital of Philadelphia
IE: Innovation Ecosystem
