# Supplementary material for: Using human factors principles to redesign a 3D lab workflow during the COVID-19 pandemic
Source: 3D Print Med. 2022 Nov 12;8:34. doi: 10.1186/s41205-022-00161-9 (PMC9655797; doi:10.1186/s41205-022-00161-9)
Supplement: Supplementary file 1 — Additional file 1. Project Submission Form. [file 41205_2022_161_MOESM1_ESM.pdf]

CHAMP Intake form

* Required

* This form will record your name, please fill your name.

1. What is the name of your project? *

2. Please describe the problem you are aiming to address *

3. How would you categorize your project? *

- ◯ Surgical Planning
- ◯ Training or Phantom
- ◯ Education
- ◯ Device or tool

CHAMP Intake

Clinical and Surgical Planning models should be request through EPIC request form to maintain the HIPPA data. Please contact silvestro@chop.edu for more support.

Thank You

CHAMP Intake

4. What testing, regulatory, and/or sterility testing or questions need to be answered to validate the integrity of this product? *

- ◯ Fit test
- ◯ User test
- ◯ Material testing
- ◯ FDA approval
- ◯ Regulatory
- ◯ Sterility
- ◯ PPDC
- ◯ Due care testing
- ◯ Other

CHAMP Intake

5. What will this project be used for? *

- ◯ Clinical
- ◯ Research
- ◯ Publication
- ◯ Crisis response

6. CHAMP aims to promote and expand the field of medical additive manufacturing through Papers, Intellectual Property, and Grants, and Supply Chain breakdowns and/or crisis response needs (PIGS). What is this project's PIGS goal? Choose as many as you'd like: *

- ◯ Research Paper
- ◯ Intellectual Property (Patents)
- ◯ Grant Existing
- ◯ Grant Targeting
- ◯ Supply Chain breakdown - filling an urgent need
- ◯ None, Help me!
- ◯ Other

7. What are the existing solutions/attempted solutions to this problem? *

8. Who is relevant to the deployment of this device and/or needs to approve this? *

9. How many copies of this product will be needed? *

10. Who else (other teams, groups, individuals) might benefit from this product? *

11. Is the progress of your project contingent on the CHAMP product you are requesting? *

- ◯ Yes
- ◯ No, the CHAMP product is just supporting the project

CHAMP Intake

12. Please explain, and list any urgent deadlines associated with this project:
*

CHAMP Intake

13. How might this request lead to potential future funding (e.g. from CHOP, PPDC, NSF, NIH, etc.)? *

14. Please provide a contact e-mail. *

15. Please attach any initial design(s) you have here:

⤒ **Upload file**

File number limit: 5 Single file size limit: 10MB Allowed file types: Word, Excel, PPT, PDF, Image, Video, Audio

This content is neither created nor endorsed by Microsoft. The data you submit will be sent to the form owner.

Microsoft Forms
